# Long-term prognosis after endoscopic submucosal dissection for esophageal cancer in older adult patients

**DOI:** 10.1186/s12876-024-03234-7

**Published:** 2024-05-14

**Authors:** Hirona Konishi, Yuji Urabe, Takeo Nakamura, Kazuki Ishibashi, Junichi Mizuno, Motomitsu Fukuhara, Takeshi Takasago, Hidenori Tanaka, Akiyoshi Tsuboi, Ken Yamashita, Yuichi Hiyama, Hidehiko Takigawa, Takahiro Kotachi, Ryo Yuge, Akira Ishikawa, Shiro Oka

**Affiliations:** 1 Department of Gastroenterology, Hiroshima University Hospital, Hiroshima, Japan; 2 Department of Gastrointestinal Endoscopy and Medicine, Hiroshima University Hospital, 1-2-3, Kasumi, Minamiku, Hiroshima, 734-8551 Japan; 3 Department of Clinical Research Center, Hiroshima University Hospital, Hiroshima, Japan; 4 Department of Molecular Pathology, Graduate School of Biomedical and Health Sciences, Hiroshima University, Hiroshima, Japan

**Keywords:** Esophageal cancer, Older individual, Endoscopic submucosal dissection, American Society of anesthesiologists classification of physical status class, Long-term prognosis

## Abstract

**Background:**

The validity of endoscopic submucosal dissection (ESD) for esophageal squamous cell carcinoma (ESCC) in older individuals with comorbidities remains unclear. Therefore, this study evaluated the safety and efficacy of ESD and additional treatment for ESCC in older adult patients.

**Methods:**

The clinicopathological characteristics and clinical outcomes of 398 consecutive older adult patients (≥ 65 years) with 505 lesions who underwent ESD for ESCC at the Hiroshima University Hospital between September 2007 and December 2019 were retrospectively evaluated. Additionally, the prognoses of 381 patients who were followed up for > 3 years were assessed.

**Results:**

The mean patient age and procedure time were 73.1 ± 5.8 years and 77.1 ± 43.5 min, respectively. The histological en bloc resection rate was 98% (496/505). Postoperative stenosis, perforation, pneumonia, and delayed bleeding were conservatively treated in 82 (16%), 19 (4%), 15 (3%), and 5 (1%) patients, respectively. The 5-year overall and disease-specific survival rates were 78.9% and 98.0%, respectively (mean follow-up time: 71.1 ± 37.3 months). Multivariate analysis showed that age and the American Society of Anesthesiologists classification of physical status class ≥III (hazard ratio: 1.27; 95% confidence interval: 1.01–1.59, *p* = 0.0392) were independently associated with overall survival. A significantly lower overall survival rate was observed in the high-risk follow-up group than in the low-risk follow-up and high-risk additional treatment groups (*p* < 0.01). However, no significant difference in disease-specific survival was observed among the three groups.

**Conclusions:**

ESD is safe for ESCC treatment in patients aged ≥ 65 years. However, additional treatments should be considered based on the patient’s general condition.

**Supplementary Information:**

The online version contains supplementary material available at 10.1186/s12876-024-03234-7.

## Introduction

Esophageal squamous cell carcinoma (ESCC) is one of the most common malignant tumors in East Asia [1]. In Japan, the peak age for ESCC onset is the 60s, with most cases occurring in individuals in their 50s and 70s [2]. Recently, Japan has had an increasingly aging population, resulting in an increasing incidence of ESCC among older individuals [3].

Generally, physiological functions and activities of daily living (ADL) decline as patients age, usually limiting their treatment options for cancer [4]. Physiological functions, ADL, and basic diseases in older adult patients vary widely among older individuals, and treatments should be considered based on the expected life prognosis, living environment, and social background. Furthermore, many patients with ESCC have underlying diseases, such as chronic obstructive pulmonary and cardiovascular diseases, because ESCC occurs mostly in regular smokers and drinkers [5].

Chemoradiotherapy (CRT) and surgical resection are usually used to treat ESCC. However, CRT frequently causes lung damage, and cardiac and pulmonary functions affect the ability to tolerate surgery. Surgery-related mortality is reportedly 2% in thoracic ESCC surgery [6] and is expected to be even higher in older adult patients and those with underlying comorbidities. Therefore, some cases of ESCC are difficult to treat using surgery or CRT [7, 8].

Endoscopic submucosal dissection (ESD) has been reported to be useful in treating ESCC, and it is considered a treatment option for patients with ESCC. However, the indication for ESD in ESCC is T1 cancer without lymph node metastasis. Additional treatment with surgery or CRT is required in cases with positive vertical margins or the risk of lymph node metastasis after ESD [9]. However, additional treatment may be difficult in patients with superficial ESCCs who require additional treatment with CRT or surgery after ESD owing to their age or underlying disease. Furthermore, determining the safety and prognosis after ESD for these patients is crucial for developing treatment strategies for superficial ESCCs in older individuals and patients with underlying diseases. Therefore, this study aimed to investigate the safety and prognosis of ESD in older adult patients with ESCC, considering their age and physical condition.

## Methods

### Study design and population

Consecutive patients with superficial ESCCs aged ≥ 65 years who underwent ESD at Hiroshima University Hospital between September 2007 and December 2019 were retrospectively enrolled. Prognoses and recurrence among patients who did not regularly visit our hospital were surveyed via telephone.

The following variables were investigated to determine the clinical outcomes of ESD: (i) complete en bloc resection, (ii) mean procedure time, and (iii) complications (perforation, postoperative bleeding, pneumonia, and postoperative stenosis). Older individuals were defined as those aged ≥ 65 years [10]. Individuals aged 65–74 and ≥ 75 years were defined as early- and late-term older adult persons, respectively, since physical function is believed to be deteriorating in late-term older adults [11]. Therefore, we divided the age group by 75 years to investigate the prognosis.

### ESD procedure

The number of Lugol-voiding lesions per endoscopic view was counted, and the grading was classified into the following categories: (i) grade A, no lesions; (ii) grade B, 1–9 lesions; and (iii) grade C, ≥ 10 lesions [12].

Esophageal ESD was performed by gastrointestinal endoscopists affiliated with the Japanese Society of Gastroenterological Endoscopy who had performed > 100 ESD procedures. High-frequency electrocautery was performed using ESG100 (Olympus, Tokyo, Japan) or VIO300D (ERBE Elektromedizin GmbH, Tubingen, Germany). An upper gastrointestinal endoscope with a water-delivery function (GIF-Q260J or GIF-H290T; Olympus Optical Industries Corporation, Tokyo, Japan) and a transparent hood attachment (TOP Corporation, Tokyo, Japan) were used. ESD was performed using DualKnife/DualKnife J and SB knife Jr after marked dots were placed outside the lesion margins using iodine staining. The injection solution was a 10% glycerin solution containing a small amount of indigo carmine for full incisions. A solution of 0.4% sodium hyaluronate (Muco Up; Boston Scientific, Tokyo, Japan) diluted twice with a 10% glycerin solution containing a small amount of indigo carmine was used for submucosal injection. The procedure time was defined as the time from the initial mucosal injection to the completion of resection. Postoperative bleeding was defined as bleeding that required transfusion or resulted in decreased hemoglobin levels by 2 g/dL within > 24 h after the procedure. Perforation was diagnosed if endoscopically confirmed during the procedure or if mediastinal emphysema or a small amount of free air was observed during chest computed tomography (CT). Stenosis was defined as the failure of a conventional single-channel endoscope to pass through the stenosis. Bleeding was endoscopically stopped using hemostatic forceps (Coagrasper, Olympus, Tokyo, Japan), and transfusion was performed as required. In cases with perforation, the mucosal defect was completely closed using EZ clips (Olympus) endoscopically, if possible. The patients were allowed to skip meals and take antibiotics until their fever and abdominal pain resolved and inflammatory findings improved. Patients with pneumonia were allowed to skip meals and take antibiotics until their fever resolved and the inflammatory findings improved. For patients with more than three-quarters circumference resection of the esophagus, a localized steroid injection was administered after ESD to prevent stenosis.

Repeated endoscopic balloon dilation was performed in patients with postoperative stenosis.

### Histopathologic evaluation

The specimens resected using ESD were stretched, pinned, and fixed in a 10% formalin solution. They were sliced at 2-mm-thick intervals and evaluated microscopically. The depth of the submucosa was determined following the General Rules for Clinical and Pathological Studies on Cancer of the Esophagus, outlined by the Japanese Society for the Esophagus [13]. Lesions were classified as T1a (epithelial [EP]/lamina propria mucosal [LPM]/muscularis mucosal [MM]) or T1b (submucosal [SM]) carcinomas. Lymphovascular invasion was assessed using only hematoxylin and eosin staining until October 2013 and subsequently with Elastica van Gieson and D2-40, in addition to hematoxylin and eosin. Curative resection is not clearly defined in the Japanese Esophageal Association guidelines [13, 14]; therefore, it was defined as T1a carcinoma with negative lymphovascular status in this study. Moreover, curative and noncurative resection cases were classified as the low-risk follow-up and high-risk groups, respectively. The complete en bloc resection rate was defined as a one-piece resection of the entire lesion with endoscopically and pathologically negative margins. Furthermore, the degree of submucosal fibrosis was classified into the none, mild, and severe groups based on a previous report [15].

### Follow-up schedule after ESD

Patients underwent annual upper gastrointestinal endoscopy after curative resection. Those who underwent additional surgery after noncurative resection also had medical examinations every 3 months postoperatively. CT examinations and upper gastrointestinal endoscopy were performed every 6 months and annually, respectively. Moreover, patients who received follow-up treatment or CRT after noncurative resection also underwent upper gastrointestinal endoscopy 1–2 months after ESD, and ulcer scars were confirmed after excision. Subsequently, upper gastrointestinal endoscopy was performed every 4–6 months, while CT was conducted every 4–6 months to evaluate lymph node metastasis, distant metastasis, and recurrence. However, the surveillance period was flexible depending on each patient’s physical condition. Recurrence was confirmed based on imaging or pathological findings. Local residual recurrence was defined as the scar recurrence after ESD. Death due to ESCC was defined as primary cancer death, and death from other causes as deaths due to other diseases.

### Variables investigated

Variables for clinical outcomes of ESD were investigated as follows: complete en bloc resection, average procedure time, and adverse events (postoperative stenosis, perforation, pneumonia, and delayed bleeding). We analyzed the risk factors for poor prognosis and compared overall survival (OS) and disease-specific survival (DSS) according to the risk factors for poor prognosis among the low-risk follow-up, high-risk additional treatment, and high-risk follow-up groups.

The American Society of Anesthesiologists classification of physical status (ASA-PS) [16] was used for categorizing the preoperative physical status of patients as follows: (i) ASA-PS class I, normal healthy patients; (ii) ASA-PS class II, patients with mild systemic disease; (iii) ASA-PS class III, patients with a severe systemic disease that is not life-threatening; (iv) ASA-PS class IV, patients with extreme systemic disorders that have become an imminent threat to life regardless of the type of treatment; (v) ASA-PS class V, moribund patients who are not expected to survive; and (vi) ASA-PS class VI, patients declared brain-dead whose organs are being removed for donor purposes. Prognostic nutritional indexes were also evaluated, including the Onodera Prognostic Nutritional Index (PNI = 10 albumin (Alb) [g/dL] + 0.005 total lymphocyte count [/mm^3^ peripheral blood]) [17], neutrophil-to-lymphocyte ratio (NLR = total lymphocyte count [/mm^3^ peripheral blood]/total neutrophil count [/mm^3^ peripheral blood]) [18], Geriatric Nutritional Risk Index (GNRI = 14.89 Alb [g/dL] + 41.7 weight [kg]/22 height^2^ [m^2^]) [19], Alb, and body mass index (= weight [kg]/height^2^ [m^2^]).

### Ethical statement

This study protocol was conducted in accordance with the principle of the Declaration of Helsinki and was approved by the Institutional Review Board of Hiroshima University (approval number: E2023-0195). All patients were informed of the risks and benefits of ESD and provided written informed consent.

### Statistical analysis

Quantitative data are presented as mean ± standard deviation or percentage. Differences in categorical variables were analyzed using the chi-square test with the Yates correction or Fisher’s exact test. The risk factors for poor prognosis were analyzed using univariate and multivariate analyses. Continuous and qualitative variables were analyzed using Student’s *t*-test or the Mann–Whitney U test and Pearson’s chi-squared test, respectively. Statistical significance was set at *p* < 0.05. Logistic regression analysis was performed to examine the risk factors for poor OS. OS and DSS rates were calculated using the Kaplan–Meier method. All statistical analyses were performed using JMP statistical software version 16.0.0 (SAS Institute, Cary, North Carolina, USA).

## Results

### Patient and lesion characteristics

Among the consecutive patients with superficial ESCC who underwent ESD, 398 older adult patients with 505 superficial lesions were enrolled. Prognoses and recurrences were evaluated in 381 older adult patients (96%). Supplementary Tables 1 and 2 present the clinicopathological characteristics of the patients.

### ESD outcomes

Table 1 presents the short-term outcomes. The mean procedure time, en bloc resection rate, and complete en bloc resection rate were 77.1 min, 98%, and 95%, respectively. Severe fibrosis was observed in 86 (17%) lesions. Endoscopic balloon dilatation was performed in 82 (16%) patients because of postoperative stenosis. Additionally, perforation, pneumonia, and delayed bleeding were observed in 19 (4%), 15 (3%), and 5 (1%) patients, respectively. All cases were resolved with conservative treatment, and no ESD-related deaths occurred. Pathological diagnoses included 380 (75%), 61 (12%), and 64 (13%) EP/LPM, MM, and SM lesions, respectively. In total, 44 (9%) and 22 (4%) lesions had positive lymphatic invasion and venous invasion, respectively. Noncurative resections using ESD (lymphovascular invasion positive and/or pT1b-SM) were observed with 76 (15%) lesions. Supplementary Table 3 presents the clinical characteristics of lesions and short-term outcomes by ASA-PS. Tumor size, procedure time, pathological diagnosis, and lymphovascular involvement were significantly different between the ASA-PS classes I/II and III.

**Table 1:** Outcomes of endoscopic submucosal dissection

Variables		(%)
Procedure time, min, mean ± SD	77.1 ± 43.5	
En bloc resection (%)	496	(98)
Complete en bloc resection (%)	479	(95)
Submucosal fibrosis (%)		
None/mild	419	(83)
Severe	86	(17)
Adverse event (%)		
Postoperative stenosis	82	(16)
Perforation	19	(4)
Pneumonia	15	(3)
Delayed bleeding	5	(1)
Pathological diagnosis (%)		
EP/LPM	380	(75)
MM	61	(12)
SM	64	(13)
Lymphovascular involvement* (%)	54	(11)
Ly1	44	(9)
V1	22	(4)

EP, epithelial; LPM, lamina propria mucosae; MM, musclaris mucosae; SM, submucosa; Ly, lymphatic invasion; V, venous invasion.

* overlapped.

### Prognoses after ESD

The prognoses of 381 (96%) patients were investigated (mean follow-up period of 71.1 ± 37.3 months). Among the 76 patients diagnosed with noncurative resection based on the pathological findings from ESD specimens, 40, 7, 1, and 6 were additionally treated with CRT, radiotherapy (RT), chemotherapy, and surgery, respectively, and 22 were followed up without additional treatment.

Supplementary Table 4 shows the causes of death in these patients. In total, 104 patients died during the observation period; seven of them died because of ESCC. Four of the seven patients were diagnosed with an invasive depth of pT1a without lymphovascular invasion based on the pathological findings after ESD and were followed up without additional treatment. The diagnoses were pT1a-EP, pT1a-LPM, and pT1a-MM in one, one, and two cases, respectively (the pT1a-EP and pT1a-LPM in one case each were performed before October 2013). Lymph node metastasis from the metachronous carcinoma, which was 0–IIa in the cervix and diagnosed as cT1b-SM, recurred in the patient diagnosed with pT1a-EP, while lymph node metastasis from the primary carcinoma recurred in the other patients. Contrastingly, three of the seven patients who died because of ESCC were diagnosed with invasive depth pT1a with lymphovascular invasion or pT1b based on the pathological findings after ESD. Two of the three patients were treated with CRT after ESD, and the other patient was followed up without additional treatment.

The most common cause of death from other diseases was cancer, excluding esophageal cancer, pneumonia, and cardiac disease. The breakdown of deaths due to other cancers was as follows: lung cancer, *n* = 7 (19%); oral cancer, *n* = 6 (16%); liver cancer, *n* = 4 (11%); colorectal cancer, *n* = 4 (11%); pancreatic cancer, *n* = 4 (11%); bladder cancer, *n* = 3 (8%); gastric cancer, *n* = 2 (5%); malignant lymphoma cancer, *n* = 2 (5%); myelodysplastic syndromes, *n* = 2 (5%); duodenal cancer, *n* = 1 (3%); renal cancer, *n* = 1 (3%); and occult primary cancer, *n* = 1 (3%). Overall, 24 of the 37 patients had a history of other organ cancers, and we performed ESD because their cancers were believed to be under control. However, 15 patients died because of the recurrence of their cancers. Two of the 37 patients were diagnosed with other organ cancers for which they were planned to undergo curative surgery simultaneously with ESCC. Although both patients underwent surgery for other cancers after ESD for ESCC, they died owing to uncontrolled cancer of other organs. Subsequently, 11 of the 37 patients died of newly found cancer of other organs > 2 years after ESD for ESCC.

Table 2 presents the OS according to the prognostic factors. Univariate analysis with log-rank tests showed that age, ASA-PS, Alb, PNI, GNRI, NLR, tumor location, and history of advanced cancer, excluding esophageal cancer, were significantly associated with impaired survival. However, multivariate analysis showed that age (hazard ratio: 1.02; 95% confidence interval (CI): 1.00–1.04, *p* = 0.0458) and ASA-PS class III (hazard ratio: 1.27; 95% CI 1.01–1.59, *p* = 0.0392) were independently associated with OS.

**Table 2:** Risk factors associated with poor overall survival

Variables	Univariate analysis				Multivariate analysis		
	HR	95% CI	*P*-value		HR	95% CI	*P*-value
Age, year	1.03	1.01–1.05	0.0008		1.02	1.00-1.04	0.0458
Sex			0.5844				
male	1.09	0.80–1.48					
female	1	Ref					
BMI	0.97	0.93-1.00	0.0616				
ASA-PS			0.0051				0.0392
Class I/II	1	Ref			1	Ref	
Class III	1.37	1.10–1.70			1.27	1.01–1.59	
Alb, g/dL	0.58	0.45–0.75	< 0.0001		0.83	0.47–1.46	0.5167
PNI	0.95	0.93–0.97	< 0.0001		0.99	0.95–1.04	0.7303
GNRI	0.97	0.96–0.99	< 0.0001		0.99	0.97-1.00	0.1161
NLR	0.64	0.40–0.99	< 0.0001		0.90	0.52–1.56	0.7155
Use of anticoagulants and/or antiplatelet drugs	1.21	0.93–1.56	0.1535				
History of other advanced cancer	1.35	1.09–1.67	0.0052		1.27	1.01–1.58	0.0794
History of esophageal cancer	1.09	0.84–1.40	0.5344				
Multiple Lugol voiding lesion			0.2018				
A/B	1	Ref					
C	1.15	0.93–1.41					
Tumor size, mm	1.00	1.00-1.01	0.3082				
Macroscopic type							
0-IIa	1	Ref					
0-IIb	0.70	0.35–1.40	0.3121				
0-IIc	1.32	0.82–2.12	0.2600				
Tumor location							
Ce or Ut	1	Ref			1	Reference	
Mt	0.64	0.49–0.84	0.0010		0.66	0.50–0.87	0.0032
Lt or Ae	0.93	0.58–1.26	0.6339		0.96	0.70–1.31	0.7874
Circumferential range							
<2/3	1	Ref					
≧2/3	0.95	0.70–1.27	0.7057				
Whole circumference	1.22	0.77–1.95	0.3963				
Postoperative stricture	0.91	0.70–1.18	0.4746				

Three hundred and eighty-one elderly patients with ESCC were observed for > 3 years. The overall survival rate was examined for each risk factor using a log-rank test. Multivariate analysis was calculated using a Cox proportional hazards model

BMI, body mass index; ASA-PS, American Society of Anesthesiologists classification of physical status; PNI, prognostic nutritional index; GNRI, geriatric nutritional risk index; NLR, neutrophil to lymphocyte ratio; Ce, cervical esophagus; Ut, upper thoracic esophagus; Mt, mid-thoracic esophagus; Lt, lower thoracic esophagus; Ae, abdominal esophagus

**Fig. 1:**
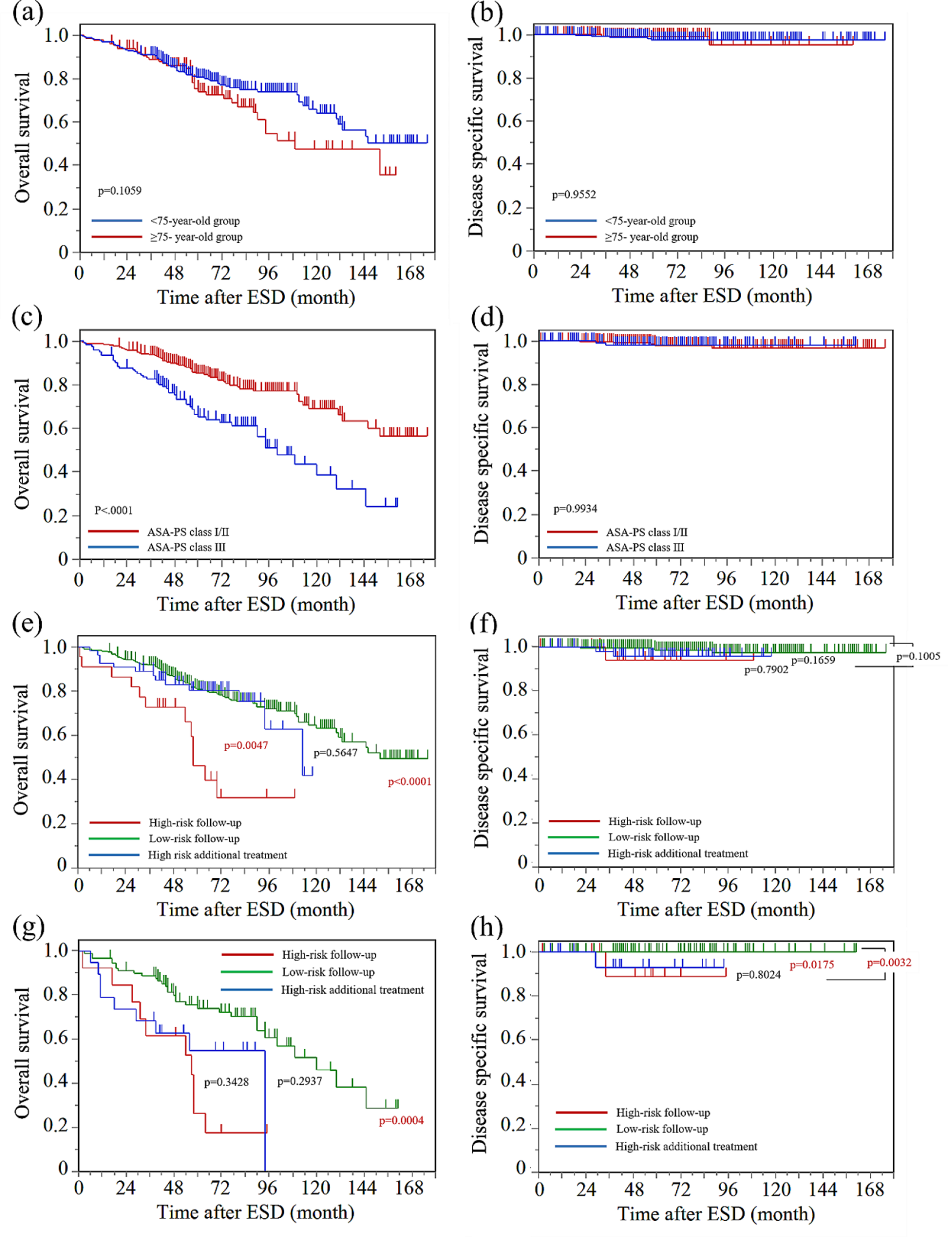
Overall and disease-specific survival (OS and DSS) rates in 381 older adult patients with superficial ESCC. (**a**, **b**) OS and DSS according to age. No difference in OS and DSS was observed between the < 75-year-old and ≥ 75-year-old groups. (**c**) OS according to the ASA-PS classification. A significantly lower OS rate was observed in patients in the ASA-PS class III than in those in the ASA-PS class I/II (*p* < 0.0001). (**d**) DSS according to the ASA-PS classification. No difference was observed between patients in the ASA-PS classes I/II and III. (**e**) OS according to the presence or absence of additional treatments after ESD. A significantly lower OS rate was observed in the high-risk follow-up group than in the low-risk follow-up and high-risk additional treatment groups (*p* < 0.01). (**f**) DSS according to the presence or absence of additional treatments after ESD. No significant difference was observed between the high-risk follow-up, low-risk follow-up, and high-risk additional treatment groups. (**g**, **h**) DSS according to the presence or absence of additional treatments among patients in the ASA-PS class III. A significantly lower DSS rate was observed in the high-risk group than in the low-risk group (*p* < 0.05), and no difference in DSS rate was observed between the high-risk additional treatment and the high-risk follow-up group

Figure 1 shows the OS and DSS rates of older adult patients with ESCC after ESD. No difference in OS and DSS rates was observed between the < 75-year-old and ≥ 75-year-old groups (Fig. 1a and b). The OS rate in the ASA-PS class III was significantly lower than that in the ASA-PS class I/II (*p* < 0.0001), although no difference in DSS rate was observed between the two groups (Fig. 1c and d). A significantly lower OS rate was observed in the high-risk follow-up group than in the low-risk follow-up and high-risk additional treatment groups (*p* < 0.01). However, no significant difference was observed in DSS among the three groups (Fig. 1e and f). A significantly lower DSS rate was observed in the high-risk group of patients in the ASA-PS class III than in the low-risk group of patients in the ASA-PS class III (*p* < 0.05). Furthermore, no difference was observed in DSS between the high-risk additional treatment and high-risk follow-up groups (Fig. 1g and h).

## Discussion

This study revealed that no ESD-related deaths occurred among patients with ESCC aged ≥ 65 years, and age and ASA-PS were significantly associated with poor prognostic factors. Additionally, no significant difference was observed in the prognoses between the presence or absence of additional treatment in the high-risk groups of patients in the ASA-PS class III, which is a poor prognostic factor. Previous studies reported no differences in short-term ESD outcomes between older and younger patients [12, 20, 21]. Meanwhile, the postoperative 30- and 90-day mortality rates were significantly higher in older adult patients (≥ 70 years) than in younger patients (< 70 years) [22]. Previous studies [23, 24] reported significantly more postoperative adverse events in older adult patients than in younger patients. Additionally, comparing the short- and long-term outcomes of endoscopic versus surgical therapy for early ESCC in older adult patients showed that the 2-year survival rate after endoscopy was significantly higher than that after surgery [25]. Therefore, ESD for ESCC is considered appropriate and a safe and minimally invasive treatment for older adult patients.

Our study’s data showed that age and the ASA-PS were poor prognostic factors in older adult patients with ESCC after ESD. Previous studies [26–30] have examined the association between nutritional and general health statuses and prognosis in older adult patients with cancer. However, only a few studies have investigated the relationship between nutritional status and prognosis after ESD for ESCC [31]. Therefore, we hypothesized that patients’ nutritional and general health statuses, such as their ASA-PS and tumor grade, are associated with prognoses in older adult patients who underwent ESD for ESCC. Consequently, this study’s findings show that nutritional status correlated with prognosis in the univariate analysis and not independently in the multivariate analysis, indicating that the ASA-PS and age were associated with prognosis. The findings of previous studies and our study differed because ESD is a minimally invasive treatment compared to surgery, and ESD treatment is relatively unlikely to contribute to the worsening of nutritional status or decline in ADL compared to surgery. Therefore, the ASA-PS can be considered a simple and useful index to indicate poor prognosis in patients after ESD for ESCC.

In this study, treatment methods were selected based on the physicians’ decision, considering the patient’s general condition and aspirations. Therefore, the results in Supplementary Table3 indicate that patients in poor general condition tended to be selected for ESD, a minimally invasive treatment, even in cases of cT1b-SM. Additionally, the procedure time might have been significantly longer in the ASA-PS class III cases than in the ASA-PS class I/II cases because they had more lesions that were larger and deeper in tumor size and depth, respectively.

The most common cause of death from other diseases was cancer, excluding esophageal cancer, pneumonia, and cardiac disease. This suggests that patients who could not undergo additional surgery based on the physicians’ decision may have had poorer prognoses than other patient groups. Moreover, in patients in the ASA-PS class III, which is a poor prognostic factor, the presence or absence of additional treatment after ESD in the high-risk group did not result in differences in the prognoses. This may be because (i) surgical resection was avoided and CRT, RT, or chemotherapy was opted for in ASA-PS class III cases, considering the patient’s general condition; (ii) treatments were performed with less than the usual dose at the physicians’ discretion; (iii) patients lacked sufficient survival time to benefit from additional treatments; and (iv) from previous reports, patients with poor general health and numerous comorbidities, such as those in the ASA-PS class III, particularly older adults, were more prone to treatment-related adverse events [7, 8]. In addition, although ASA-PS class III cases had significantly larger tumor diameters, longer procedure times, a higher proportion of pT1b-SM cases, and a higher proportion of lymphatic cases than ASA-PS class I/II cases (Supplementary Table 3), there was no significant difference in DSS between ASA-PS class I/II and III cases (Fig. 1d). Therefore, ESD can be safely performed in older adult patients. Moreover, if the pathological diagnosis after ESD is curative, the death of patients with ESCC may be prevented, regardless of their general condition. However, follow-up without additional treatment after ESD may be considered acceptable for patients in the high-risk group with poor general conditions.

This study had some limitations. First, this was a single-center, retrospective study rather than a multicenter study. The concept of sarcopenia related to muscle mass is reportedly useful as a prognostic factor in older individuals [32]. However, because this is a retrospective study, we did not measure grip strength, tests related to physical function, and skeletal muscle mass before ESD to assess the presence of sarcopenia. Therefore, we could not evaluate its relevance to the results of this study. Second, no comparison was made between older and younger patients. Third, the common criteria for deciding which treatment is better, as an additional treatment or follow-up, are unclear, and the decision is left to the attending physician’s discretion. Fourth, central pathological reviews were not performed. Although experienced pathologists evaluated the patients according to the guidelines, variations in the histologic diagnoses may exist. Finally, no exact method exists for the surveillance of ESCC after ESD in older adults, and it varies slightly based on the attending physician’s discretion.

In conclusion, our study showed that ESD was a safe and effective treatment and prevented ESCC-related death in older adult patients regardless of age and ASA-PS class ≤ III. Furthermore, to limit additional treatment after ESD to only older high-risk cases, a list of high-risk cases with rapid progression to additional treatment after ESD should be narrowed down using pathological and genetic analyses.

### Electronic supplementary material

Below is the link to the electronic supplementary material.

Supplementary Material 1

## Data Availability

All data generated during this study are included in this article. Further inquiries can be directed to the corresponding author.

## Abbreviations

ESD: Endoscopic submucosal dissection
ASA-PS: American Society of Anesthesiologists classification of physical status
ESCC: Esophageal squamous cell carcinoma
ADL: Activities of daily living
CRT: Chemoradiotherapy
CT: Computed tomography
EP: Epithelial
LPM: Lamina propria mucosal
MM: Muscularis mucosal
SM: Submucosal
PNI: Onodera Prognostic Nutritional Index
NLR: Neutrophil-to-lymphocyte ratio
GNRI: Geriatric Nutritional Risk Index
RT: Radiotherapy
CI: Confidence interval
OS: Overall survival
DSS: Disease-specific survival

## References

[1] Arnold M, Soerjomataram I, Ferlay J (2015). Global incidence of oesophageal cancer by histological subtype in 2012. Gut.

[2] Watanabe M, Toh Y, Ishihara R (2023). Comprehensive registry of esophageal cancer in Japan, 2015. Esophagus.

[3] Fitzmaurice C, Dicker D, Pain A (2015). The global burden of Cancer 2013. JAMA Oncol.

[4] Van Deudekom FJ, Klop HG, Hartgrink HH (2018). Functional and cognitive impairment, social functioning, frailty and adverse health outcomes in elderly patients with esophageal cancer, a systematic review. J Geriatr Oncol.

[5] Oze I, Charvat H, Matsuo K (2019). Revisit of an unanswered question by pooled analysis of eight cohort studies in Japan: does cigarette smoking and alcohol drinking have interaction for the risk of esophageal cancer?. Cancer Med.

[6] Motoyama S, Yamamoto H, Miyata H (2020). Impact of certification status of the institute and surgeon on short-term outcomes after surgery for thoracic esophageal cancer: evaluation using data on 16,752 patients from the National Clinical Database in Japan. Esophagus.

[7] Schweigert M, Solymosi N, Dubecz A (2013). Current outcome of esophagectomy in the very elderly: experience of a German high-volume center. Am Surg.

[8] Lu X, Wu H, Wang J (2014). Short- and long-term outcomes of definitive chemoradiotherapy in patients with esophageal carcinoma aged ≥ 75 years. Mol Clin Oncol.

[9] Japan Esophageal Society (2017). Japanese classification of Esophageal Cancer, 11th Edition: part I. Esophagus.

[10] OECD: Elderly population (indicator). 2023. https://data.oecd.org/pop/elderly-population.htm. Accessed 29 November 2023.

[11] Ouchi Y, Rakugi H, Arai H (2017). Redefining the elderly as aged 75 years and older: proposal from the Joint Committee of Japan Gerontological Society and the Japan Geriatrics Society. Geriatr Gerontol Int.

[12] Katada C, Yokoyama T, Yano T (2016). Alcohol consumption and multiple dysplastic lesions increase risk of squamous cell carcinoma in the esophagus, head, and neck. Gastroenterology.

[13] Kitagawa Y, Ishihara R, Ishikawa H (2023). Esophageal cancer practice guidelines 2022 edited by the Japan esophageal society: part 1. Esophagus.

[14] Mizumoto T, Hiyama T, Oka S (2018). Curative criteria after endoscopic resection for superficial esophageal squamous cell carcinomas. Dig Dis Sci.

[15] Matsumoto A, Tanaka S, Oba S (2010). Outcome of endoscopic submucosal dissection for colorectal tumors accompanied by fibrosis. Scand J Gastroenterol.

[16] Keats AS (1978). The ASA classification of physical status—a recapitulation. Anesthesiology.

[17] Onodera T, Goseki N, Kosaki G (1984). Prognostic nutritional index in gastrointestinal surgery of malnourished cancer patients. Nihon Geka Gakkai Zasshi.

[18] Yamanaka T, Matsumoto S, Teramukai S (2007). The baseline ratio of neutrophils to lymphocytes is associated with patient prognosis in advanced gastric cancer. Oncology.

[19] Bouillanne O, Morineau G, Dupont C (2005). Geriatric nutritional risk index: a new index for evaluating at-risk elderly medical patients. Am J Clin Nutr.

[20] Song BG, Min YW, Lee JH (2017). Efficacy and safety of endoscopic submucosal dissection in elderly patients with esophageal squamous cell carcinoma. Surg Endosc.

[21] Matsumoto Y, Kimura K, Zhou Q (2019). Treatments and outcomes of elderly patients with esophageal cancer: comparison with younger patients. Mol Clin Oncol.

[22] Laurent A, Marechal R, Farinella E (2022). Esophageal cancer: outcome and potential benefit of esophagectomy in elderly patients. Thorac Cancer.

[23] Morita M, Egashira A, Yoshida R (2008). Esophagectomy in patients 80 years of age and elderly with carcinoma of the thoracic esophagus. J Gastroenterol.

[24] Cijs TM, Verhoef C, Steyerberg EW (2010). Outcome of esophagectomy for cancer in elderly patients. Ann Thorac Surg.

[25] Cummings LC, Kou TD, Schluchter MD (2016). Outcomes after endoscopic versus surgical therapy for early esophageal cancers in an elderly population. Gastrointest Endosc.

[26] Kakiuchi Y, Kuroda S, Kikuchi S (2022). Prognostic risk factors for postoperative long-term outcomes in elderly stage IA gastric cancer patients. J Gastrointest Oncol.

[27] Zhang B, Li Y, Chen Y (2022). Prognosis-related nutritional score for Cancer patients (PRNS): a clinical nutritional score derived from a retrospective cohort study. J Transl Med.

[28] Yoshifuku Y, Oka S, Tanaka S (2016). Long-term prognosis after endoscopic submucosal dissection for early gastric cancer in super-elderly patients. Surg Endosc.

[29] Nishimura T, Oka S, Tanaka S (2021). Long-term prognosis after endoscopic submucosal dissection for colorectal tumors in patients aged over 80 years. BMC Gastroenterol.

[30] Dang C, Wang M, Qin T (2022). How can we better predict the prognosis of patients with pancreatic cancer undergoing surgery using an immune-nutritional scoring system?. Surgery.

[31] Iwai N, Dohi O, Yamada S (2022). Prognostic risk factors associated with esophageal squamous cell carcinoma patients undergoing endoscopic submucosal dissection: a multi-center cohort study. Surg Endosc.

[32] Fang P, Zhou J, Xiao X (2023). The prognostic value of Sarcopenia in oesophageal cancer: a systematic review and meta-analysis. J Cachexia Sarcopenia Muscle.

