# Supplementary material for: Long-term prognosis after endoscopic submucosal dissection for esophageal cancer in older adult patients
Source: BMC Gastroenterol. 2024 May 14;24:164. doi: 10.1186/s12876-024-03234-7 (PMC11091999; doi:10.1186/s12876-024-03234-7)
Supplement: Supplementary file 1 — Supplementary Material 1 [file 12876_2024_3234_MOESM1_ESM.docx]

**Supplementary tables**

**Table 1 Clinical characteristics of patients treated with endoscopic submucosal dissection**

| **Variables** |  | **(%)** |
| --- | --- | --- |
| Age, year, mean ± SD | 73.1±5.8 | |
| Sex, male (%) | 346 | (87) |
| BMI, kg/m^2^, mean ± SD | 21.8±2.9 | |
| ASA-PS (%) |  |  |
| Class I/II | 271 | (68) |
| Class III | 127 | (32) |
| Alb, g/dL, mean ± SD | 4.2±0.4 | |
| PNI, mean ± SD | 50.5±5.6 | |
| GNRI, mean ± SD | 104.5±8.7 | |
| NLR, mean ± SD | 0.5±0.2 | |
| Comorbidities* (%) |  |  |
| Hypertension | 139 | (35) |
| Chronic kidney disease | 83 | (21) |
| Cardiovascular disease | 74 | (19) |
| Diabetes mellitus | 62 | (16) |
| Cerebral vascular disease | 35 | (9) |
| Liver cirrhosis | 9 | (2) |
| Arteriosclerosis obliterans | 4 | (1) |
| Use of antithrombotic drugs (%) | 75 | (19) |
| History of esophageal cancer* (%) | 75 | (19) |
| Lesion treated by endoscopic resection | 57 | (76) |
| Lesion treated by CRT (RT) | 22 | (15) |
| Lesion treated by surgery | 11 | (9) |
| History of other advanced cancer (%) | 142 | (36) |
| Multiple Lugol voiding lesion (%) |  |  |
| A/B | 254 | (64) |
| C | 144 | (36) |

SD, standard deviation; BMI, body mass index; ASA-PS, American society of Anesthesiologists classification of physical status; PNI, prognostic nutritional index; GNRI, geriatric nutritional risk index; NLR, neutrophil to lymphocyte ratio; CRT, chemoradiotherapy; RT, radiation therapy

* overlapped

**Table 2 Clinical characteristics of lesions treated with endoscopic submucosal dissection**

| **Variables** |  | **(%)** |
| --- | --- | --- |
| Tumor size, mm, mean ± SD | 25.9±17.4 | |
| Macroscopic type (%) |  |  |
| 0-Ⅱa | 19 | (4) |
| 0-Ⅱb | 19 | (4) |
| 0-Ⅱc | 467 | (92) |
| Tumor location (%) |  |  |
| Ce/Ut | 97 | (19) |
| Mt | 278 | (55) |
| Lt/Ae | 130 | (26) |
| Circumferential range (%) |  |  |
| <2/3 | 424 | (84) |
| ≥2/3 | 60 | (12) |
| Whole circumference | 21 | (4) |
| Clinical diagnosis (%) |  |  |
| EP/LPM | 382 | (76) |
| MM/SM1 | 96 | (19) |
| SM2 | 27 | (5) |

SD, standard deviation; Ce, cervical esophagus; Ut, upper thoracic esophagus; Mt, mid-thoracic esophagus; Lt, lower thoracic esophagus; Ae, abdominal esophagus; EP, epithelial; LPM, lamina propria mucosae; MM, musclaris mucosae; SM, submucosa

**Table 3 Clinical characteristics of lesions treated with endoscopic submucosal dissection and short-term outcomes**

| **Variables** | **ASA-PS class I/II**  **n=354** | | **ASA-PS class III**  **n=151** | | **P value** |
| --- | --- | --- | --- | --- | --- |
| Tumor size, mm, mean ± SD | 24.9±0.9 | | 28.4±1.4 | | 0.0408 |
| Macroscopic type (%) |  |  |  |  | 0.0821 |
| 0-Ⅱa | 9 | (3) | 10 | (7) |  |
| 0-Ⅱb | 12 | (3) | 7 | (4) |  |
| 0-Ⅱc | 333 | (94) | 134 | (89) |  |
| Tumor location (%) |  |  |  |  | 0.8501 |
| Ce/Ut | 66 | (19) | 31 | (20) |  |
| Mt | 195 | (55) | 83 | (55) |  |
| Lt/Ae | 93 | (26) | 37 | (25) |  |
| Circumferential range (%) |  |  |  |  | 0.8035 |
| <2/3 | 300 | (85) | 124 | (82) |  |
| ≥2/3 | 41 | (11) | 19 | (13) |  |
| Whole circumference | 13 | (4) | 8 | (5) |  |
| Procedure time, min, mean ± SD | 74.4±2.3 | | 83.5±3.5 | | 0.0302 |
| En bloc resection (%) | 349 | (99) | 147 | (97) | 0.3363 |
| Complete en bloc resection (%) | 339 | (96) | 140 | (93) | 0.1560 |
| Submucosal fibrosis (%) |  |  |  |  | 0.4827 |
| None/mild | 291 | (82) | 128 | (85) |  |
| Severe | 63 | (18) | 23 | (15) |  |
| Adverse event (%) |  |  |  |  |  |
| Postoperative stenosis | 56 | (16) | 26 | (17) | 0.6963 |
| Perforation | 12 | (3) | 5 | (3) | 0.9643 |
| Pneumonia | 8 | (2) | 7 | (5) | 0.1499 |
| Delayed bleeding | 2 | (1) | 3 | (2) | 0.1396 |
| Pathological diagnosis (%) |  |  |  |  | 0.0023 |
| EP/LPM | 278 | (79) | 102 | (68) |  |
| MM | 43 | (12) | 18 | (12) |  |
| SM | 33 | (9) | 31 | (20) |  |
| Lymphovascular involvement* (%) | 29 | (8) | 25 | (17) | 0.0054 |
| Ly1 | 24 | (7) | 20 | (13) | 0.0183 |
| V1 | 9 | (3) | 13 | (9) | 0.0022 |

SD, standard deviation; Ce, cervical esophagus; Ut, upper thoracic esophagus; Mt, mid-thoracic esophagus; Lt, lower thoracic esophagus; Ae, abdominal esophagus; EP, epithelial; LPM, lamina propria mucosae; MM, musclaris mucosae; SM, submucosa; Ly, lymphatic invasion; V, venous invasion

**Table 4 Cause of death in patients**

| **Variables** |  | **(%)** |
| --- | --- | --- |
| Primary cancer death (recurrence) | 7 | (7) |
| Death due to other diseases | 97 | (93) |
| Other cancer | 37 | (38) |
| Lung cancer | 7 | (19) |
| Oral cancer | 6 | (16) |
| Liver cancer | 4 | (11) |
| Colorectal cancer | 4 | (11) |
| Pancreatic cancer | 4 | (11) |
| Bladder cancer | 3 | (8) |
| Gastric cancer | 2 | (5) |
| Malignant lymphoma | 2 | (5) |
| Myelodysplastic syndromes | 2 | (5) |
| Duodenal cancer | 1 | (3) |
| Renal cancer | 1 | (3) |
| Occult primary cancer | 1 | (3) |
| Pneumonia | 21 | (22) |
| Heart disease | 7 | (7) |
| Others | 32 | (33) |
| Other etiologies | 17 | (53) |
| Etiology unknown | 15 | (47) |

This table shows a summary for cause of death in 104 ESCC patients who died among followed-up periods. Others include that patients were the cause of death is not clear.
